# Altered Frequency-Dependent Brain Activation and White Matter Integrity Associated With Cognition in Characterizing Preclinical Alzheimer’s Disease Stages

**DOI:** 10.3389/fnhum.2021.625232

**Published:** 2021-02-16

**Authors:** Siyu Wang, Jiang Rao, Yingying Yue, Chen Xue, Guanjie Hu, Wenzhang Qi, Wenying Ma, Honglin Ge, Fuquan Zhang, Xiangrong Zhang, Jiu Chen

**Affiliations:** ^1^Institute of Neuropsychiatry, The Affiliated Brain Hospital of Nanjing Medical University, Fourth Clinical College of Nanjing Medical University, Nanjing, China; ^2^Fourth Clinical College of Nanjing Medical University, Nanjing, China; ^3^Institute of Brain Functional Imaging, Nanjing Medical University, Nanjing, China; ^4^Department of Rehabilitation, The Affiliated Brain Hospital of Nanjing Medical University, Nanjing, China; ^5^Department of Psychosomatics and Psychiatry, The Affiliated ZhongDa Hospital, School of Medicine, Southeast University, Nanjing, China; ^6^Department of Radiology, The Affiliated Brain Hospital of Nanjing Medical University, Nanjing, China; ^7^Department of Neurology, The Affiliated Brain Hospital of Nanjing Medical University, Nanjing, China; ^8^Department of Psychiatry, The Affiliated Brain Hospital of Nanjing Medical University, Nanjing, China; ^9^Department of Geriatric Psychiatry, The Affiliated Brain Hospital of Nanjing Medical University, Nanjing, China

**Keywords:** amplitude of low-frequency fluctuation, diffusion tensor imaging, amnestic mild cognitive impairment, subjective cognitive decline, non-amnestic mild cognitive impairment

## Abstract

**Background:**

Subjective cognitive decline (SCD), non-amnestic mild cognitive impairment (naMCI), and amnestic mild cognitive impairment (aMCI) are regarded to be at high risk of converting to Alzheimer’s disease (AD). Amplitude of low-frequency fluctuations (ALFF) can reflect functional deterioration while diffusion tensor imaging (DTI) is capable of detecting white matter integrity. Our study aimed to investigate the structural and functional alterations to further reveal convergence and divergence among SCD, naMCI, and aMCI and how these contribute to cognitive deterioration.

**Methods:**

We analyzed ALFF under slow-4 (0.027–0.073 Hz) and slow-5 (0.01–0.027 Hz) bands and white matter fiber integrity among normal controls (CN), SCD, naMCI, and aMCI groups. Correlation analyses were further utilized among paired DTI alteration, ALFF deterioration, and cognitive decline.

**Results:**

For ALFF calculation, ascended ALFF values were detected in the lingual gyrus (LING) and superior frontal gyrus (SFG) within SCD and naMCI patients, respectively. Descended ALFF values were presented mainly in the LING, SFG, middle frontal gyrus, and precuneus in aMCI patients compared to CN, SCD, and naMCI groups. For DTI analyses, white matter alterations were detected within the uncinate fasciculus (UF) in aMCI patients and within the superior longitudinal fasciculus (SLF) in naMCI patients. SCD patients presented alterations in both fasciculi. Correlation analyses revealed that the majority of these structural and functional alterations were associated with complicated cognitive decline. Besides, UF alterations were correlated with ALFF deterioration in the SFG within aMCI patients.

**Conclusions:**

SCD shares structurally and functionally deteriorative characteristics with aMCI and naMCI, and tends to convert to either of them. Furthermore, abnormalities in white matter fibers may be the structural basis of abnormal brain activation in preclinical AD stages. Combined together, it suggests that structural and functional integration may characterize the preclinical AD progression.

## Introduction

Subjective cognitive decline (SCD), amnestic mild cognitive impairment (aMCI), and non-amnestic mild cognitive impairment (naMCI) are regarded to be at high risk of converting to Alzheimer’s disease (AD) (Petersen et al., 2001, 2009; Jessen et al., 2014). SCD and aMCI are characterized as preclinical AD stages (Jessen et al., 2014; Rabin et al., 2017; Hao et al., 2019). While multiple research papers regard naMCI as part of the disease continuum due to diverse aspects (Yang et al., 2018; Xue C. et al., 2019), some have reservations (Vos et al., 2013, 2015; Wilcockson et al., 2019). Accordingly, the utilization of combined structural and functional neuroimaging analyses is required to further reveal convergence and divergence among normal controls (CN), SCD, naMCI, and aMCI and assist in characterizing the preclinical AD progression.

Resting-state functional magnetic resonance imaging (rs-fMRI) is applied for AD pathophysiologic measurement through blood-oxygen-level dependent (BOLD) signal alterations (Golestani et al., 2017; Kuang et al., 2019; Xue J. et al., 2019). Amplitude of low-frequency fluctuations (ALFF), defined by BOLD signal fluctuations under low frequency bands, is widely utilized in preclinical AD detection to reflect intrinsic neuronal activities (Lu et al., 2007; Hare et al., 2017). To note, the selection of specific frequency bands is critical for the precise reflection of neuronal activity (Biswal et al., 1995; Fox and Raichle, 2007; Han et al., 2011), and accurate detection of regional deterioration (Buzsaki and Draguhn, 2004). Previous studies have consistently indicated that the slow-5 band (0.01–0.027 Hz) and slow-4 band (0.027–0.073 Hz) are more sensitive in reflecting the compensation and deterioration of AD (Liu et al., 2014; Yang et al., 2018, 2019). Thus, we infer that ALFF under slow-4 and slow-5 bands may be a splendid choice for revealing preclinical AD spontaneous neuronal activities. Furthermore, diffusion tensor imaging (DTI) detects water molecule diffusion and is sensitive in white matter atrophy detection (Nir et al., 2013). DTI-based measures, e.g., fractional anisotropy (FA), mean diffusivity (MD), and relative anisotropy (RA), have been widely proven to be correlated with MCI and AD symptoms (Chandra et al., 2019; Dumont et al., 2019; Zhang et al., 2019). Therefore, utilization of DTI analyses may assist in uncovering alterative characteristics of preclinical AD stages from a structural perspective.

Numerous research papers have utilized ALFF calculation and DTI analyses separately in revealing typical characteristics of prodromal AD stages. For ALFF detection, abnormal ALFF values were widely detected in the frontal, occipital, and temporal lobes in aMCI patients and were proven to act as a sensitive biomarker for AD pathology (Han et al., 2011; Ren et al., 2016). Also, gradual disturbances among CN, SCD, aMCI, and AD were revealed through correlation analyses between ALFF alterations and neuropsychological assessments (Yang et al., 2018). DTI tractography may assist in distinguishing significantly deteriorated white matter integrity and serve as biomarkers for MCI (Teipel et al., 2019; Zhang et al., 2019). SCD suffered from more subtle alterations than MCI, indicating a potential disease progression sequence (Brueggen et al., 2019). Furthermore, white matter alterations were also revealed to be correlated with progressed cognitive decline (Power et al., 2019). Through MRI modalities, which included combined ALFF and DTI analyses, Gupta et al. created a classification tool to distinguish converting MCI (which progresses to AD) from non-converting MCI (Gupta et al., 2020). However, the mentioned research lacked consideration of the entire AD preclinical stages (i.e., SCD, naMCI, and aMCI) and analyses of the relationship with cognition. For the ALFF calculation part, separate sub-divided frequency bands were regarded to better present impairment (Buzsaki and Draguhn, 2004). Besides, comprehensive cognitive domains may reflect real cognitive conditions more accurately (Beauchet et al., 2015; Ramanan et al., 2017). A combination of structural and functional neuronal analyses may reveal alterations from a comprehensive perspective and help better understand disease pathology and progression.

This study analyzed DTI indices and ALFF to measure structural and functional alterations in preclinical AD stages. Next, correlation analyses were utilized in matched groups among cognition, white matter integrity, and ALFF values to further reveal internal associations. We speculated that convergence and divergence existed among SCD, naMCI, and aMCI. Moreover, relations between structural and functional alterations may further be revealed under AD progression.

## Materials and Methods

### Subjects

Our study gained approval from the responsible Human Participants Ethics Committee of the Affiliated Nanjing Brain Hospital (Nos. 2018-KY010-01 and 2020-KY010-02) (Nanjing, China). Every participant provided written informed consent.

Data applied in this study were obtained from our in-house database: Nanjing Brain Hospital-Alzheimer’s Disease Spectrum Neuroimaging Project (NBH-ADsnp) (Nanjing, China), which is constantly being updated. The details of the NBH-ADsnp-related information are provided in Supplementary Material. A total of 79 elderly patients were recruited initially from hospitals and communities through advertisement and broadcasting. Twelve of the participants were excluded due to no MRI data (*n* = 10) and excessive head motion (cumulative translation or rotation >3.0 mm or 3.0°, *n* = 2). A total of 67 patients were eventually included after exclusion with accordance to previous studies (Chen et al., 2016a; Dillen et al., 2017; Xue C. et al., 2019). Detailed inclusive and exclusive criteria are summarized in Supplementary Material.

### Neuropsychological Assessments

Evaluation of the four cognitive domains (i.e., visuospatial function, executive function, information processing speed, and episodic memory) was conducted based on neuropsychological assessments according to previous studies (Chen et al., 2016b; Xue C. et al., 2019). Division and evaluation details are listed in Supplementary Material.

### MRI Data Collection

Detailed MRI data acquisitive parameters of the NBH-ADsnp database are provided in Supplementary Material.

### ALFF Data Analyses

For ALFF analyses, data preprocessing was performed by MATLAB2013b^1^ and DPABI (Yan et al., 2016). Briefly, the preprocessing steps included slice-timing and head motion correction, nuisance covariate regression, spatial normalization, spatial smoothing with a Gaussian kernel of 6 mm × 6 mm × 6 mm (full width at half maximum, FWHM) (Chen et al., 2016b, 2020), and detrending. Participants with excessive head motion (cumulative translation or rotation >3.0 mm or 3.0°) were excluded. The details regarding preprocessing steps are provided in the Supplementary Material.

We used DPABI to calculate the multi-band ALFF values after the image preprocessing procedure. In brief, we used a Fast Fourier Transform (FFT) to convert the time series into the frequency domain, and obtained the power spectrum, the square root of which was calculated and averaged across frequency intervals that were pre-defined. The averaged square root obtained by the procedure was then taken as ALFF. ALFF was z-normalized as Fisher’s z transformation ALFF (zALFF) after the calculation (Alonso-Solis et al., 2017). According to previous studies (Han et al., 2011; Liu et al., 2014; Yang et al., 2018, 2019), we chose the slow-4 band and slow-5 band out of the five bands, discarding the other three frequency bands (i.e., slow-2, slow-3, and slow-6) due to the reflection of high-frequency physiological noise, white matter signals, and very low frequency drift (Han et al., 2011).

### DTI Data Analyses

We applied the Pipeline for Analyzing Brain Diffusion Images toolkit (PANDA,^2^), combining the FMRIB Software Library (FSL,^3^) and Diffusion toolkit for DTI data preprocessing. The brief procedures included: (a) image conversion from DICOM to NIFTI; (b) brain extraction; (c) brain mask estimation; (d) image cropping and realignment; (e) eddy current and motion correction; (f) acquisition averaging; and (g) diffusion tensor and scalar measures (i.e., FA, MD, and RA) calculation.

### Statistical Analysis

Statistical analyses were conducted by the Statistical Package for the Social Sciences (SPSS) software version 22.0 (IBM, Armonk, New York, United States). Comparison of demographic and neuropsychological data within CN, SCD, naMCI, and aMCI was done by analysis of covariance (ANCOVA) and the chi-square test (for gender). We performed two-sample *T*-tests for *post hoc* comparisons between any two groups (Bonferroni-corrected, *p* < 0.05).

The DPABI software was used to compare differences in ALFF indices among the four groups. Differences under slow-4 and slow-5 bands were revealed by conducting ANCOVA after controlling for age, gender, education level, and GM volume. A non-parametric permutation test (1,000 permutations) was applied to get control of the false positive rate in the cluster-level inference (Smith and Nichols, 2009). We corrected multiple comparisons of statistical maps to a significant level of *p* < 0.05, with a cluster size of over 1,350 mm^3^. We used two-sample *T*-tests to investigate the differences between every two groups under the significant regions detected in the ANCOVA test after controlling for age, gender, education level, and GM volume. A strict TFCE-FWE was applied to correct the results to a significant level of *p* < 0.05, with a cluster size of over 270 mm^3^.

In the DTI analyses, for each metric (i.e., FA, MD, and RA), we performed multiple comparisons between any two matched groups. False discovery rate (FDR) correction was utilized at a threshold of *p* < 0.05.

To note, various neurocognitive assessments were divided into four cognitive domains as mentioned above (see Supplementary Material for details). We obtained the composite Z scores of each domain by transforming raw scores into normalized Z scores. After extracting the ROI Series using the REST software (Song et al., 2011), the relationship among altered ALFF values, abnormal white matter integrity, and composite Z scores of each cognitive domain was examined using Pearson’s correlation analysis after controlling for the effects of age, gender, and educational level.

Furthermore, all data in this study have been subjected to tests for normality. This study used the Shapiro–Wilk test to assess data normal distribution. To note, all data (age, education level, neuropsychological characteristics, altered ALFF values, and altered white matter integrity) in this study exhibited a normal/Gaussian distribution, except gender.

## Results

### Demographic and Neuropsychological Characteristics

Demographic and neuropsychological characteristics of the four groups can be found in Table 1. To begin, SCD scored the highest among the four groups in the SCD-Q test. In comparison with CN, aMCI presented significantly lower scores in MDRS-2, MMSE, and MoCA tests (all *p* < 0.05). The aMCI group exhibited significant deficits in visuospatial function, executive function, episodic memory, and information processing speed (all *p* < 0.05). The aMCI group also showed significantly lower scores in executive function and episodic memory in comparison with the SCD group (all *p* < 0.05). Compared to CN, naMCI displayed lower scores in executive function and information processing speed, while naMCI showed lower scores in executive function, information processing speed, and education level in comparison with SCD (all *p* < 0.05). aMCI displayed lower scores in episodic memory in comparison with naMCI (all *p* < 0.05) (see Table 1).

**TABLE 1:** Demographic data of CN, SCD, naMCI, and aMCI.

	CN	SCD	naMCI	aMCI	*F*-values (χ2)	*P*-values
				
	*n* = 21	*n* = 10	*n* = 15	*n* = 33		
Age (years)	57.52 ± 8.072	63.10 ± 8.774	63.87 ± 8.568	66.03 ± 8.579^*a*^	4.388	0.007
Gender (male/female)	7/14	4/6	6/9	11/22	6.696	0.010
Education level (years)	12.05 ± 2.747	13.85 ± 1.827	10.60 ± 2.694^*a*^	11.06 ± 3.358	3.086	0.032
MMSE	28.81 ± 1.209	27.70 ± 1.160	28.27 ± 1.710	26.88 ± 2.027^*a*^	6.156	0.001
MDRS	141.00 ± 2.915	138.80 ± 3.393	137.07 ± 3.432	134.82 ± 7.970^*a*^	5.193	0.003
MoCA	26.20 ± 2.624	25.00 ± 3.432	24.14 ± 2.931	22.40 ± 3.379^*a*^	5.357	0.002
**Composite Z score of each cognitive domain**						
Episodic memory	0.49 ± 0.584	0.57 ± 0.484	0.33 ± 0.532	−0.62 ± 0.549^*a,b,c*^	25.373	0.000
Information processing speed	0.45 ± 0.759	0.41 ± 0.468	−0.40 ± 0.658^*a,b*^	−0.22 ± 0.775^*a*^	6.535	0.001
Executive function	0.45 ± 0.728	0.43 ± 0.504	−0.27 ± 0.489^*a,b*^	−0.24 ± 0.613^*a,b*^	7.974	0.000
Visuospatial function	0.26 ± 0.567	0.41 ± 0.446	−0.12 ± 0.681	−0.24 ± 1.011	2.790	0.046

*Data are exhibited as mean ± standard deviation (SD). *p* < 0.05 meant significant differences existed among CN, SCD, naMCI, and aMCI. All *p*-values were achieved through ANOVA analyses except gender (chi square test). Between-group comparisons were further utilized to reveal specific alterations among matched groups (^*a*^: compared to CN; ^*b*^: compared to SCD; and ^*c*^: compared to naMCI), Bonferroni corrected, *p* < 0.05. CN, normal control; SCD, subjective cognitive decline; naMCI, non-amnestic mild cognitive impairment; aMCI, amnestic mild cognitive impairment; MMSE, Mini-Mental State Examination; MDRS, Mattis Dementia Rating Scale; MoCA, Montreal Cognitive Assessment; ANOVA, analysis of variance.*

### Altered ALFF Values Among CN, SCD, naMCI, and aMCI

**FIGURE 1:**
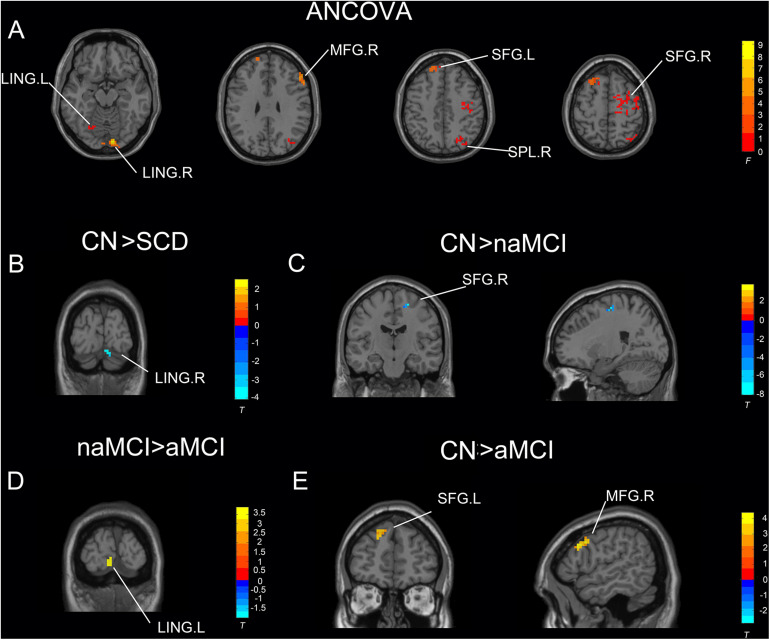
Significant differences in ALFF under the slow-4 band among CN, SCD, naMCI, and aMCI. **(A)** Results of ANCOVA analysis among CN, SCD, naMCI, and aMCI (cluster size >1,350 mm^3^, *p* < 0.05). **(B–E)** Results of voxel-wised analyzed two-sample *T*-tests (cluster size >270 mm^3^, *p* < 0.05, TFCE-FWE corrected). To note, these analyses set age, gender, educational level, and GM volume as covariates. CN, normal control; SCD, subjective cognitive decline; naMCI, non-amnestic mild cognitive impairment; aMCI, amnestic mild cognitive impairment; ANCOVA, analysis of covariance; SPL, superior parietal lobe; SFG, superior frontal gyrus; LING, lingual gyrus; MFG, middle frontal gyrus. R, right hemisphere; L, left hemisphere.

Under the slow-4 band, the ANCOVA analysis exhibited significant differences in six brain regions among CN, SCD, naMCI, and aMCI, including the right superior parietal lobule, bilateral lingual gyrus (LING), bilateral superior frontal gyrus (SFG), and right middle frontal gyrus (cluster size >1,350 mm^3^, *p* < 0.05). Compared to CN, SCD, and naMCI displayed an increase in ALFF in the right LING and left SFG, respectively. In comparison with CN, aMCI showed reduced ALFF values in the right middle frontal gyrus and left SFG. Compared to aMCI, naMCI exhibited increased ALFF values in the right LING (cluster size >270 mm^3^, *p* < 0.05, TFCE-FWE corrected) (see Table 2 and Figure 1).

**TABLE 2:** Differences in ALFF under slow-4 and slow-5 bands among CN, SCD, naMCI, and aMCI.

Brain regions (aal)		Peak MNI coordinate			*F*/*T*-values	Cluster size (mm^3^)
		
		*x*	*y*	*z*		
**Slow-4**	**ANCOVA**					
	L lingual gyrus	−27	−72	−3	0.117	1512
	R lingual gyrus	12	−90	−15	9.087	2646
	R middle frontal gyrus	57	18	33	6.876	1539
	R superior parietal lobule	27	−66	63	0.133	1890
	L superior frontal gyrus	−12	48	42	4.646	2943
	R superior frontal gyrus	36	0	36	3.465	5859
	**CN vs. SCD**					
	R lingual gyrus	6	−90	−15	–4.076	324
	**CN vs. naMCI**					
	R superior frontal gyrus	21	−15	60	–6.766	324
	**CN vs. aMCI**					
	R middle frontal gyrus	51	30	30	4.418	1107
	L superior frontal gyrus	−18	48	36	3.350	432
	**naMCI vs. aMCI**					
	L lingual gyrus	−3	−93	−6	3.898	297
**Slow-5**	**ANCOVA**					
	R superior frontal gyrus	27	60	−6	6.964	3267
	R middle frontal gyrus	30	18	48	6.885	2187
	L superior parietal lobule/L precuneus	−24	−48	66	10.854	2889
	**CN vs. aMCI**					
	R middle frontal gyrus	36	57	6	3.409	378
	**SCD vs. aMCI**					
	R superior frontal gyrus	30	18	48	4.114	1404
	**naMCI vs. aMCI**					
	L superior parietal lobule/L precuneus	−24	−48	66	5.298	1863

*All significant results were presented in control of age, gender, and educational level with *p* < 0.05. TFCE-FWE correction was applied for voxel-wised two-sample *T*-test. To note, cluster size was set over 1,350 mm^3^ in ANCOVA analyses and over 270 mm^3^ in two-sample *T*-tests. CN, normal control; SCD, subjective cognitive decline; naMCI, non-amnestic mild cognitive impairment; aMCI, amnestic mild cognitive impairment; ALFF, amplitude of low-frequency fluctuation; ANCOVA, analysis of covariance; AAL, anatomical automatic labeling; MNI, Montreal Neurological Institute; R, right; L, left.*

**FIGURE 2:**
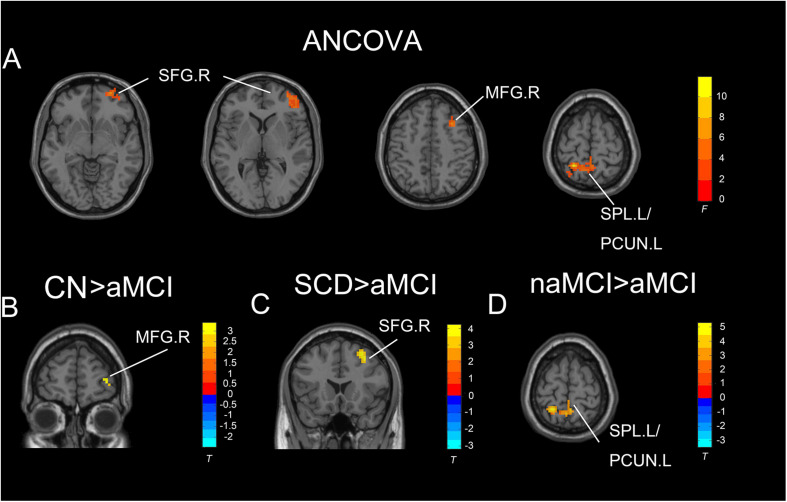
Significant differences in ALFF under the slow-five band among CN, SCD, naMCI, and aMCI. **(A)** Results of ANCOVA analysis among CN, SCD, naMCI, and aMCI (cluster size >1,350 mm^3^, *p* < 0.05). **(B–D)** Results of voxel-wised analyzed two-sample *T*-tests (cluster size >270 mm^3^, *p* < 0.05, TFCE-FWE corrected). To note, these analyses set age, gender, educational level, and GM volume as covariates. CN, normal control; SCD, subjective cognitive decline; naMCI, non-amnestic mild cognitive impairment; aMCI, amnestic mild cognitive impairment; ANCOVA, analysis of covariance; SPL, superior parietal lobe; SFG, superior frontal gyrus; PCUN, precuneus; MFG, middle frontal gyrus. R, right hemisphere; L, left hemisphere.

Under the slow-5 band, the ANCOVA analysis exhibited significant differences in three brain regions among CN, SCD, naMCI, and aMCI, including the right SFG, right middle frontal gyrus, and right superior parietal lobule/left precuneus (cluster size >1,350 mm^3^, *p* < 0.05). Compared to CN, SCD, and naMCI, aMCI exhibited descended ALFF values in the right SFG, right middle frontal gyrus, and right superior parietal lobule/left precuneus, respectively (cluster size >270 mm^3^, *p* < 0.05, TFCE-FWE corrected) (see Table 2 and Figure 2).

### Deteriorated White Matter Integrity Among CN, SCD, naMCI, and aMCI

**FIGURE 3:**
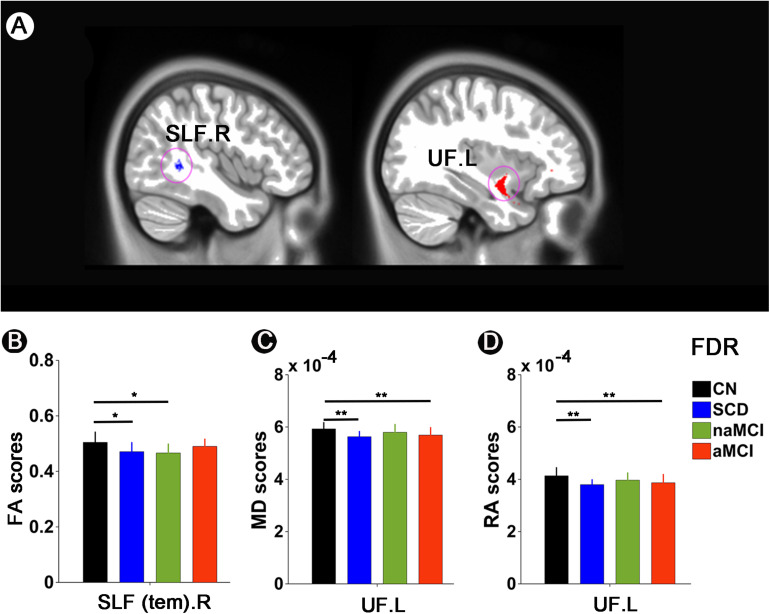
White matter integrity alterations among CN, SCD, naMCI, and aMCI. **(A,B)** FA scores in the SLF.R among CN, SCD, naMCI, and aMCI. **(A,C)** MD scores in the UF.L among CN, SCD, naMCI, and aMCI. **(A,D)** RA scores in the UF.L among CN, SCD, naMCI, and aMCI. */** meant that significance existed between paired groups. All these significant results were corrected applying FDR correction with a threshold of *p* < 0.05 after controlling for age, gender, educational level, and GM volume. CN, normal control; SCD, subjective cognitive decline; naMCI, non-amnestic mild cognitive impairment; aMCI, amnestic mild cognitive impairment. FA, fractional anisotropy; MD, mean diffusivity; RA, relative anisotropy; SLF, superior longitudinal fasciculus; UL, uncinate fasciculus. R, right; L, left.

Significant results were mainly present in the superior longitudinal fasciculus (SLF) and uncinate fasciculus (UF) among paired groups. Compared to CN, SCD, and naMCI presented significantly descended FA in the SLF.R. Besides, in comparison with CN, SCD and aMCI exhibited significantly descended MD in the UF.L. Compared to CN, SCD and aMCI also presented significantly descended RA in the UF.L (all *p* < 0.05, FDR corrected) (see Figure 3).

### Associations Between Aaltered ALFF Values, White Matter Integrity, and Cognition

**FIGURE 4:**
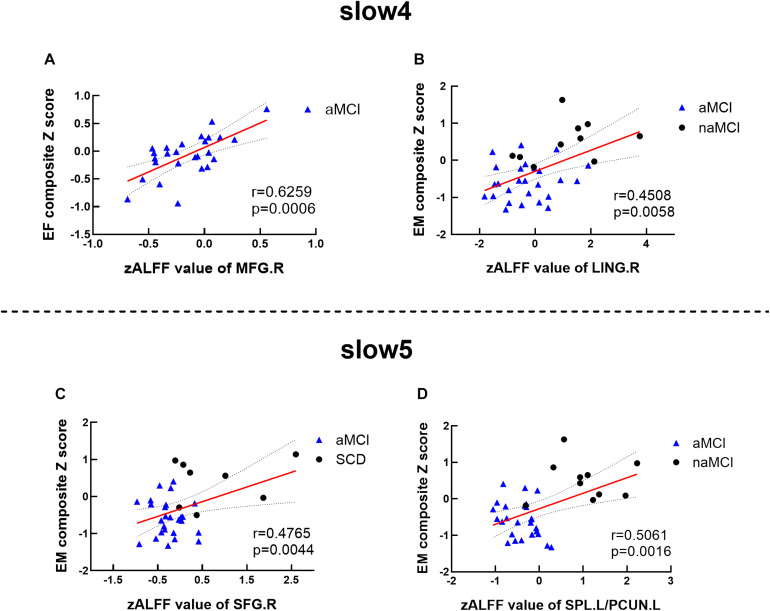
Altered ALFF values associated with cognition. **(A,B)** Significant correlations between ALFF values and cognition among matched groups under the slow-4 band. **(C,D)** Significant correlations between ALFF values and cognition among matched groups under the slow-5 band. These analyses set age, gender, educational level, and GM volume as covariates. All presented results were corrected by utilizing Bonferroni correction with a threshold of *p* < 0.05. EM, episodic memory; EF, executive function. SCD, subjective cognitive decline; naMCI, non-amnestic mild cognitive impairment; aMCI, amnestic mild cognitive impairment. MFG, middle frontal gyrus; LING, lingual gyrus; SFG, superior frontal gyrus; SPL, superior parietal lobe; PCUN, precuneus; R, right; L, left.

Significant associations between altered ALFF values and cognition were detected with age, gender, and education level as covariates (*p* < 0.05, Bonferroni corrected). Under the slow-4 band, the ALFF values of the right superior parietal lobule were positively correlated with executive function in the groups of CN and aMCI (*r* = 0.6557, *p* = 0.0002) (see Figure 4). Under the slow-5 band, the ALFF values of the right superior parietal lobule/left precuneus were positively correlated with episodic memory within the groups of naMCI and aMCI (*r* = 0.4734, *p* = 0.0035). The ALFF values of the right superior parietal lobule in combination with SCD and aMCI were positively correlated with episodic memory (*r* = 0.4866, *p* = 0.0034) (see Figure 4).

**FIGURE 5:**
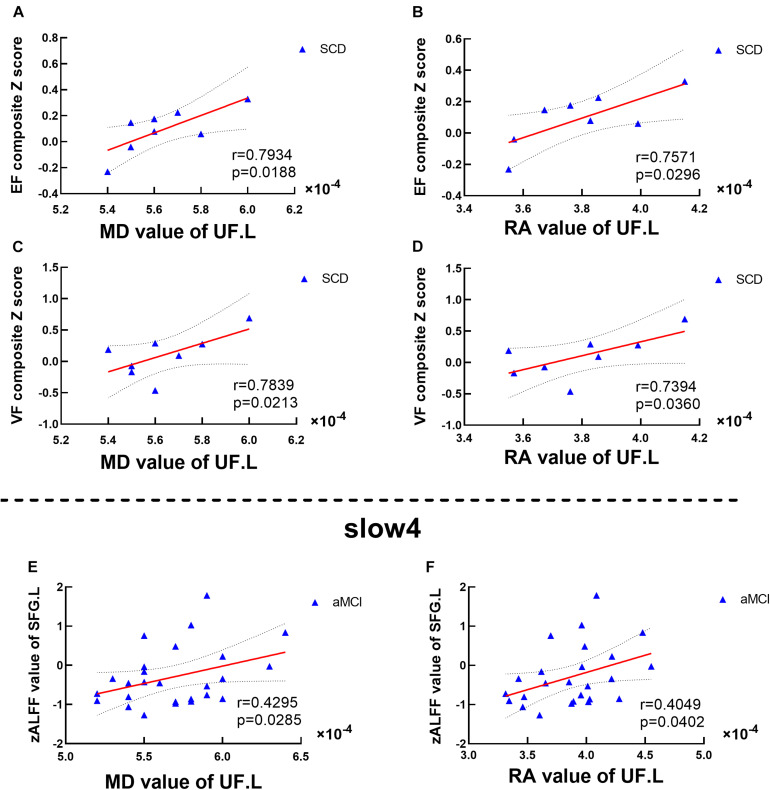
Altered DTI scores associated with abnormal cognition and ALFF values. **(A–D)** Significant correlations between altered DTI scores and cognition. **(E,F)** Significant correlations between altered ALFF values and abnormal DTI scores. This study set age, gender, educational level, and GM volume as covariates.EF, executive function; VF, visuospatial function. SFG, superior frontal gyrus; SCD, subjective cognitive decline; aMCI, amnestic mild cognitive impairment. MD, mean diffusivity; RA, relative anisotropy; UL, uncinate fasciculus. L, left.

Significant associations between altered DTI scores and cognitions were detected with age, gender, and education level as covariates (*p* < 0.05). MD values of the UF.L were positively correlated with executive function in SCD patients (*r* = 0.7934, *p* = 0.0188). RA values of the UF.L were positively correlated with executive function in SCD patients (*r* = 0.7571, *p* = 0.0296). MD values of the UF.L were positively correlated with visuospatial function in SCD patients (*r* = 0.7839, *p* = 0.0213). RA values of the UF.L were positively correlated with visuospatial function in SCD patients (*r* = 0.7394, *p* = 0.0360) (see Figure 5).

Significant associations between altered DTI scores and abnormal ALFF values were detected with age, gender, and education level as covariates (*p* < 0.05). Under the slow-4 band, MD values of the UF.L were positively correlated with ALFF values of the left SFG in aMCI patients (*r* = 0.4295, *p* = 0.0285). Besides, RA values of the UF.L were positively correlated with ALFF values of the left SFG in aMCI patients (*r* = 0.4049, *p* = 0.0402) (see Figure 5).

## Discussion

To the best of our knowledge, our study was the first to combine ALFF detection with DTI analyses and cognition to reveal the inner relationship of CN, SCD, naMCI, and aMCI. Our results discovered ascended ALFF values mainly in SCD and naMCI patients while descended ALFF values in aMCI patients in comparison with the other three groups. aMCI and naMCI patients reflected white matter alterations in the UF and SLF, respectively, while SCD patients exhibited alterations in both fasciculi. To note, the majority of damage was associated with decline in specific cognitive domains. On balance, these results indicate that SCD may act as the preclinical stage of naMCI or aMCI. Besides, aMCI has a divergent deteriorative pattern and degree compared to naMCI. Overall, this study indicates the complicated relationship between structural and functional deterioration and further implies that structural and functional integration may characterize the preclinical AD disease progression.

### Convergent Structural and Functional Alterations in SCD and aMCI Patients

As was presented, white matter integrity alterations in the UF within SCD patients were associated with deteriorated executive function and visuospatial function performance. Besides, UF white matter alterations were correlated with descended ALFF values in the SFG within aMCI patients. Descended ALFF values were also detected in the MFG within aMCI patients, which was in close association with deteriorated executive function performance. According to previous studies, the UF serves as a major white matter tract connecting the lateral orbitofrontal cortex and anterior temporal lobes bidirectionally. Due to its bidirectionality, UF alterations may result in both temporal and frontal dysfunction. Studies have discovered decision-making dysfunction and deterioration of specific learning and mnemonic acquisition associated with altered UF values (Von Der Heide et al., 2013; Hein et al., 2018). Furthermore, the MFG and SFG all belong to prefrontal brain regions which are closely connected to the UF. Lower ALFF values represent weakened spontaneous neural activity. Anatomically, white matter degeneration is associated with gray matter atrophy which is correlated with neuronal dysfunction. Our study results further imply the consistency of ALFF value deterioration and DTI alterations. Besides, the rostral area of the MFG has been revealed to be closely related with working memory and executive cognitive function (Barbey et al., 2013). MCI patients have presented executive dysfunction associated with the MFG in previous studies (Zheng et al., 2014; Xue C. et al., 2019). Combined together, it indicates that dysfunctional neuronal activities and altered white matter integrity jointly lead to executive dysfunction. The SFG is usually considered to be strongly connected with working memory (Alagapan et al., 2019). The SFG is also in association with the UF. It is reported that strong connections exist between episodic memory and UF alterations (Platel, 2005; Christidi et al., 2014). We speculate that interaction between the UF and SFG may be potential reasons for the presented results of dysfunction in episodic memory in the SFG. Notably, compared to SCD and aMCI exhibited a significant decrease in ALFF values in the SFG, which further demonstrates the continuous episodic memory decline from SCD to aMCI in patients.

Altogether, in accordance with these results, we may indicate that SCD and aMCI share convergence in the mechanism of UF alterations. Previous studies have revealed decreased FA values and increased MD values in AD patients (Mayo et al., 2018; Brueggen et al., 2019). Our study, however, discovered decreased MD values. We imply that this may reflect the potential compensatory mechanism. Decreased RA values were also detected in the UF. Few analyses focused on RA alterations in preclinical AD stages. In accordance with multiple sclerosis, Klawiter et al. (2011) discovered that RA alterations were related to demyelination. We speculate this mechanism also exists in preclinical AD stages. Through these UF white matter integrity alterations, which are associated with gray matter damage, prefrontal regions reflect dysfunctional conditions which further lead to comprehensive cognitive decline. Besides, in detail, both SCD and aMCI present similarly deteriorated cognition in episodic memory, indicating that SCD may act as a prodromal stage of aMCI (Li et al., 2016; Yan et al., 2018).

### Convergent Structural and Functional Alterations in SCD and naMCI Patients

Significantly decreased FA values were detected in both SCD and naMCI patients in the SLF. The SLF is a major white matter tract between the temporoparietal junction and parietal lobe with the frontal lobe. According to previous studies, the SLF engages in working memory regulation and somatosensory information transference (Hecht et al., 2015; Wang et al., 2016). Our study may prove the convergent deteriorative mechanism between SCD and naMCI in fasciculi. However, no descended ALFF value was detected in SCD and naMCI patients. Several previous animal studies have indicated that white matter damage appears prior to cortical plaques (Desai et al., 2010; Horiuchi et al., 2012). However, a reversed conclusion has been raised with regard to vascular insufficiency. Still, the tight correlation between white matter atrophy and neuronal dysfunction has been confirmed (Nasrabady et al., 2018). Araque Caballero et al. (2018) discovered that regional white matter degeneration occurs years before the onset of symptoms. In accordance with our results, we speculate that in the SLF, structural abnormality may be detected earlier in these two disease stages.

Besides, the LING and SFG presented increased ALFF values in SCD and naMCI patients, respectively. Ewers et al. (2011) reported that neurodegenerative pathology leads to the stimulation of hyper-excited neurons by accumulated amyloid plaques. Increased ALFF values indicate enhanced neuronal connectivity within related brain regions (Di et al., 2013; Yang et al., 2018). Furthermore, the LING belongs to the visual network that is associated with visual processing (Bogousslavsky et al., 1987). The SFG belongs to the prefrontal cortex (PFC) which is in possession of prefrontal working memory activity (Funahashi, 2006; Riley and Constantinidis, 2015) and further predicts the performance of episodic memory (Melrose et al., 2020). Compensation and deterioration of these two regions have been widely detected in previous studies (Kirby et al., 2010; Kusne et al., 2017; Liu et al., 2017; Zhu et al., 2017). On balance, this phenomenon implies the neuronal plasticity of brain regions, indicating that ALFF may serve as an important role in early functional compensation detection.

### Divergent Structural and Functional Alterations in naMCI and aMCI Patients

Non-amnestic mild cognitive impairment patients mainly presented decreased white matter integrity in the SLF. Furthermore, ALFF analyses discovered that naMCI patients exhibited ascended ALFF values in the SFG. These indicate the consistency of brain region requisition in both deterioration and compensation aspects from structural and functional levels, respectively. This may indicate the potential damaging progression of naMCI in the SLF and connected frontal regions according to our results.

Amnestic mild cognitive impairment patients detected deteriorated ALFF values in diverse brain regions (i.e., the MFG, SFG, PCUN, and LING). For DTI analyses, white matter alterations were mostly located in the UF. We detected associations between altered white matter integrity in the UF and ALFF deterioration in the SFG. As is mentioned, we speculate that UF alterations interact with dysfunction in the SFG and MFG. Combined together, structural and functional damage leads to a sharp decline in comprehensive cognition. Descended ALFF values in the MFG are connected with executive dysfunction. Partial MFG belongs to prefrontal regions and is in involved in executive function (Zhao et al., 2016). With the UF participating in execution, we may speculate that functional intercommunication exists between white matter and gray matter.

To note, in comparison with naMCI and aMCI presented significantly descended ALFF values in the PCUN and LING. The LING participates in the visual network. Episodic memory integrates as much sensation as auditory and visual information (Wang et al., 2018). Researchers have discovered that episodic memory performance is related to the LING (Kondo et al., 2005; Stothart et al., 2015; Oishi et al., 2018). The PCUN is the putative pivotal region of default mode network (DMN), which is considered to be associated with self-related cognitive processing (Harrison et al., 2008; Spreng et al., 2009; Drzezga et al., 2011). This brain region is also regarded to have central roles in episodic memory that declines in early disease progression (Chen et al., 2017). Descended ALFF values have been detected in the PCUN in SCD, MCI, and AD (Liu et al., 2014; Huang et al., 2015; Yang et al., 2018). Our results reflect the association between episodic memory decline and functional deterioration in the LING and PCUN, indicating the potential neuronal network deficiency of aMCI. Furthermore, this further demonstrates that typical cognitive deterioration in aMCI tends to be episodic memory decline in comparison with naMCI. The LING and PCUN may be highly involved in the episodic memory deteriorative mechanism and serve as biomarkers for distinction between naMCI and aMCI.

Furthermore, no related atrophy in white matter with ALFF alterations was found in the PCUN and LING. We speculate that there is still a neuronal compensation mechanism in late aMCI, which can be reflected in the inapparent damage in DTI analyses. However, specific neuronal atrophy has formed. The severity of the disease leads to insufficient compensation, resulting in irreversible dysfunction. Based on this, we imply that early damage to the PCUN has not been well compensated, resulting in the clinical manifestations of decreased episodic memory in aMCI patients.

Overall, aMCI presents decreased white matter integrity in the UF and more severe ALFF alterations compared to naMCI. Therefore, this may indicate that aMCI and naMCI may not only share parallel relationships with distinctly declined cognition. aMCI may act as a more severe disease condition, which is a preclinical AD stage with enhanced research value.

## Limitations

Three limitations became apparent in the study and should be mentioned. First, the sample size was small, which may cause the results to be less reliable. To improve statistical power, a non-parametric permutation test (1,000 permutations) was applied to control the false positive rate in the cluster-level inference. Furthermore, our NBH-ADsnp database continuously recruits volunteers and gets updated. Once enough volunteers are gathered, we will further verify the accuracy of the conclusion. Second, individual differences existed in age as well as education levels among CN, SCD, naMCI, and aMCI. Although all these were used as covariates to maintain the accuracy and reliability of the study, follow-ups have been performed to further reduce this effect and confirm the conclusions reached today in the following studies. Finally, this study only used ALFF to reveal the underlying pathological mechanism of AD disease progression. Yang and colleagues have indicated that fractional fALFF (fALFF) can reduce the impact of respiration, cardiac action, or motion while other studies have suggested that ALFF has higher reliability than fALFF (Zuo et al., 2010; Yang et al., 2018). Indeed, both indicators have their strengths and weaknesses (Zuo et al., 2010). Furthermore, Han and colleagues have emphasized that both ALFF and fALFF are useful and sensitive indexes for the detection of the pathological mechanism of AD-related neurodegeneration (Han et al., 2011). Therefore, in the future, we will expand the sample to further verify the validity of our conclusions and analyze both ALFF and fALFF for an in-depth and comprehensive understanding of the underlying pathological mechanism of AD disease progression.

## Conclusion

Subjective cognitive decline presents the joint deteriorative characteristics of aMCI and naMCI and tends to convert to either aMCI and naMCI. Besides, aMCI has a divergent deteriorative pattern and degree compared to naMCI. Overall, this study further indicates that abnormalities in specific white matter fibers may be the structural basis of brain activation in preclinical AD stages, which may contribute to cognitive decline. Structural and functional integration can together characterize the preclinical AD disease progression.

## Data Availability Statement

The raw data supporting the conclusions of this article will be made available by the authors, without undue reservation.

## Ethics Statement

The studies involving human participants were reviewed and approved by the responsible Human Participants Ethics Committee of The Affiliated Nanjing Brain Hospital. The patients/participants provided their written informed consent to participate in this study.

## Author Contributions

XZ and JC designed the study. SW, CX, YY, GH, WQ, WM, and JR collected the data. SW, FZ, and JC analyzed the data and prepared the manuscript. All authors contributed to the article and approved the submitted version.

## Conflict of Interest

The authors declare that the research was conducted in the absence of any commercial or financial relationships that could be construed as a potential conflict of interest.

## Footnotes

1 http://www.mathworks.com/products/matlab/

2 http://www.nitrc.org/projects/panda

3 http://fsl.fmrib.ox.ac.uk/fsl
