# Supplementary material for: Altered Frequency-Dependent Brain Activation and White Matter Integrity Associated With Cognition in Characterizing Preclinical Alzheimer’s Disease Stages
Source: Front Hum Neurosci. 2021 Feb 16;15:625232. doi: 10.3389/fnhum.2021.625232 (PMC7921321; doi:10.3389/fnhum.2021.625232)
Supplement: Supplementary file 1 [file Data_Sheet_1.doc]

### Altered frequency-dependent brain activation and white matter integrity associated with cognitions in characterizing preclinical Alzheimer’s disease stages

Siyu Wang^1,9^, Jiang Rao^2,3^, Yingying Yue^4^, Chen Xue^2,5^, Guanjie Hu^2^, Wenzhang Qi^2,5^, Wenying Ma^6^, Honglin Ge^2^, Fuquan Zhang^7^, Xiangrong Zhang^2,8*^, Jiu Chen^1,9*^

^1^Institute of Neuropsychiatry, the Affiliated Brain Hospital of Nanjing Medical University, Fourth Clinical College of Nanjing Medical University, Nanjing, Jiangsu, 210029, China

^2^Institute of Brain Functional Imaging, Nanjing Medical University, Nanjing, 210029, China

^3^Department of rehabilitation, the Affiliated Brain Hospital of Nanjing Medical University, Nanjing, 210029, China

^4^Department of Psychosomatics and Psychiatry, the Affiliated ZhongDa Hospital, School of Medicine, Southeast University, Nanjing, 210009, China

^5^Department of Radiology, the Affiliated Brain Hospital of Nanjing Medical University, Nanjing, 210029, China

^6^Department of Neurology, the Affiliated Brain Hospital of Nanjing Medical University, Nanjing, 210009, China

^7^Department of Psychiatry, the Affiliated Brain Hospital of Nanjing Medical University, Nanjing, 210029, China

^8^Department of Geriatric Psychiatry, the Affiliated Brain Hospital of Nanjing Medical University, Nanjing, 210029, China

^9^Fourth Clinical College of Nanjing Medical University, Nanjing, 210029, China

***Running Title:*** Brain activity and fiber integrity across preclinical AD stages.

***Correspondence to**:

**Jiu Chen,** Institute of Neuropsychiatry, the Affiliated Brain Hospital of Nanjing Medical University, Fourth Clinical College of Nanjing Medical University, No.264, Guangzhou Road, Gulou District, Nanjing, Jiangsu, 210029, China. E-mail: [ericcst@aliyun.com](mailto:ericcst@aliyun.com).

**Xiangrong Zhang,** Department of Geriatric Psychiatry, the Affiliated Brain Hospital of Nanjing Medical University, No.264, Guangzhou Road, Gulou District, Nanjing, Jiangsu, 210029, China. Email: [drxrz@hotmail.com](mailto:drxrz@hotmail.com).

# Supporting Information

## SI methods

### NBH-ADsnp database

Data used in this study were obtained from the Nanjing Brain Hospital-Alzheimer’s Disease (AD) Spectrum Neuroimaging Project (NBH-ADsnp) database (in-home website: http://192.168.8.100) (Nanjing, China). NBH-ADsnp was derived from an AD Spectrum Neuroimaging Project that was launched in January 2018 by the Institute of Brain Functional Imaging, the Affiliated Brain Hospital of Nanjing Medical University (Nanjing, China). Prof. Jiu Chen, PhD, MD, from the Affiliated Brain Hospital of Nanjing Medical University, served as the principal investigator of NBH-ADsnp. NBH-ADsnp was initiated by Dr. Jiu Chen and Dr. Xiangrong Zhang and was named by Dr. Jiu Chen's research group (discussed by Chen Xue, Guanjie Hu, Wenwen Xu, Wan Liu, Wenzhang Qi, Siyu Wang, Jiani Xu, Shanshan Chen, and finally verified by Jiu Chen and Xiangrong Zhang). NBH-ADsnp is an observational study, which includes cross-sectional and longitudinal follow-up components. The goal of NBH-ADsnp was to identify early neuroimaging biomarkers of preclinical AD spectrum {subjective cognitive decline (SCD), amnestic mild cognitive impairment (aMCI), amnestic mild cognitive impairment (naMCI), and AD}, to predict disease progression of individuals within the preclinical AD spectrum, and to provide imaging-based targets for individualized intervention to prevent disease deterioration from preclinical stages to the eventually progressed AD. Initially, several hundreds of elderly individuals in NBH-ADsnp, who were all Han Chinese and right-handed, were recruited from hospitals and local communities by advertising and by means of broadcasting. This database used a standardized clinical evaluation protocol that included a medical history interview, neurologic examination, a battery of neurocognitive assessments, and a resting-state MRI scan (T1, T2, 3D T1, DTI, and BOLD) for all participants (healthy controls (CN), SCD, naMCI, aMCI, and AD). All subjects and their study partners completed the informed consent process, and study protocols were reviewed and approved by the responsible Human Participants Ethics Committee of the Affiliated Brain Hospital of Nanjing Medical University (No. 2018-KY010-01, No. 2020-KY010-02, and No. ChiCTR1900022287).

The general eligibility, inclusion, and exclusion criteria for NBH-ADsnp subjects were as follows:

Inclusion criteria of SCD subjects were identified as meeting the published SCD research criteria a proposed by the Subjective Cognitive Decline Initiative (SCD-I) , and detailed inclusion criteria were described in our previously published study , as follows: (a) self-reported persistent memory decline, which was confirmed by an informant; (b) Subjective Cognitive Decline Questionnaire (SCD-Q) score > 5 ; (c) performance within the normal range on MMSE and MoCA (adjusted for age and education); (d) Clinical Dementia Rating (CDR) = 0; and (e) subjects aged between 50 and 80 years old.

Inclusion criteria of naMCI subjects were as described in previous studies : a) normal overall cognitive function just like aMCI patients; b) tests scores about memory function in the normal range, while deficits were present in other cognitive domains, including visual spatial function, executive function, language function, and information processing speed; and (c) subjects aged between 50 and 80 years old.

Inclusion criteria of aMCI subjects were as per the diagnostic criteria defined by Peterson et al. as well as following the revised consensus standards presented by Winblad et al. , and detailed inclusion criteria were described in our previously published studies : (a) memory complaint preferably corroborated by an informant or the subject for more than 3 months; (b) objective memory impairment adjusted for age and educational level; (c) normal general cognitive function of MMSE score equal or above 24; (d) no or minimal impairment in daily living activities; (e) CDR=0.5; (f) subjects aged between 50 and 80 years old; and (g) absence of dementia symptoms that were not sufficient to meet the criteria of the National Institute of Neurological and Communicative Disorders and Stroke or the AD and Related Disorders Association criteria for AD.

Inclusion criteria of CN subjects were as follows: (a) without memory complaints; (b) normal cognitive performance matched for age and education; (c) CDR=0; (d) MMSE ≥ 26; and (e) subjects aged between 50 and 80 years old .

Detailed exclusion criteria for all subjects were described in our previously published studies : (a) a past history of stroke (modified Hachinski Ischemic Scale Score of > 4), alcoholism, head injury, brain tumors, Parkinson’s disease, epilepsy, encephalitis, major depression (excluded by HAMD), or other neurological or psychiatric illness (excluded by clinical assessment and case history); (b) major medical illness (e.g., cancer, anemia, thyroid dysfunction, syphilis, or HIV); (c) severe visual or hearing loss; (d) unable to complete neuropsychological tests or with a contraindication for MRI, and (5) T2-weighted MRI showing major changes in white matter (WM), infarction, or other lesions (two experienced radiologists analyzed the scans). None of the patients used any medications.

### Subjects

The details regarding the exclusive criteria as follows: (a) subjects with no MRI data (n=10), (b) subjects with excessive head motion (>3.0mm or >3.0 degree) (n=2). The remaining patients also needed to meet the following criteria: (a) secondary school education or above, (b) no history of diseases that contribute to the degradation of brain function such as stroke, cerebral infraction, traumatic brain injury (TBI) or other neurologic or psychiatric disorders, (c) no medication history of psychotropic substances, (d) no severe loss of vision and hearing, (e) no major medical diseases (e.g., hyperthyroidism, hypothyroidism ,and cancer)

### Neuropsychological Assessments

The standard neurological assessments that used for the inclusion criteria for CN includes: Auditory Verbal Learning Test (AVLT); Logical Memory Test (LMT); Rey Complex Figure Test (CFT); Category Verbal Fluency Test (CVFT); Digit Span Test (DST); Trail Making Test (TMT-A and TMT-B); Stroop A; Stoop B; Stoop C; Digit Symbol Substitution Test (DSST); Clock-Drawing Test (CDT); Mini-Mental State Examination (MMSE); Activities of Daily Living (ADL); Mattis Dementia Rating Scale-2 (MDRS-2); Montreal Cognitive Assessment (MoCA); Subjective Cognitive Decline Questionnaire (SCD-Q); Clinical Dementia Rating (CDR); Hachinski Ischemic Scale (HIS) and Hamilton Depression Scale (HAMD).

To increase statistical power by reducing random variability, this study composited the neuropsychological tests into four cognitive domains ,which were episodic memory (EM), executive function (EF), information processing speed (IPS), and visuospatial function (VF). The details regarding the neuropsychological assessments are provided in previous studies ***.*** For EM, Auditory Verbal Learning Test (AVLT) -20-min DR, Logical Memory Test (LMT)-20-min DR, and Rey Complex Figure Test (CFT)-20-min DR were obtained. For EF, Category Verbal Fluency Test (CVFT), Digit Span Test (DST) -backward, part B of Trail Making Test (TMT-B), Stroop C, and Semantic Similarity Test were acquired. For IPS, Digit Symbol Substitution Test (DSST), part A of Trail Making Test (TMT-A), Stroop A, and Stroop B were applied. For VF, CFT and Clock-Drawing Test (CDT) were achieved.

Besides, some other neuropsychological assessments were undergone for the exclusion, categorization and evaluation of general cognitive function of the subjects, including Mini-Mental State Examination (MMSE); Activities of Daily Living (ADL); Mattis Dementia Rating Scale-2 (MDRS-2); Montreal Cognitive Assessment (MoCA); Subjective Cognitive Decline Questionnaire (SCD-Q); Clinical Dementia Rating (CDR); Hachinski Ischemic Scale (HIS) and Hamilton Depression Scale (HAMD).

### MRI Data Acquisition

All patients were imaged with a 3.0 Tesla MR Verio Siemens scanner with a standard 8-channel head coil in Affiliated Nanjing Brain Hospital. Cushions and earplugs were applied to reduce subject motion and scanner noise.

An echo-planar imaging (EPI) was used to collect resting-state functional images and the scanning parameters were as follows: repetition time (TR) = 2000 ms, echo time (TE) = 30 ms, flip angle (FA) = 90°, number of slices = 36, thickness = 4.0 mm, gap = 0 mm, matrix = 64×64, field of view (FOV) = 220 mm×220 mm, acquisition bandwidth = 100 kHz, voxel size = 3.4×3.4×4 mm^3^. 8 minutes were taken to finish the whole procedure.

High-resolution T1-weighted axial images covering the whole brain were acquired by 3D magnetization prepared rapid gradient echo (MPRAGE) sequence as follows: TR = 1900 ms, TE = 2.48 ms, inversion time (TI) = 900 ms, number of slices = 176, thickness = 1.0 mm, gap = 0.5 mm, matrix = 256×256, FA = 9°, FOV = 256 mm×256 mm, voxel size = 1×1×1 mm^3^. 4.26 minutes were taken to finish the whole procedure.

Spin echo-echo planar imaging (SE-EPI) sequences were applied to acquire DTI data. The scanning parameters were as follows: TR=10000 ms, TE=90 ms, number of slices=67, thickness=2.5 mm, gap=0 mm, matrix=128×128, FOV=256mm×256mm. Diffusion sensitizing gradient were utilized along 30 noncollinear directions (b= 1000 s/mm^2^) with a reference image without diffusion weighing (b=0 s/mm^2^). 10.57 minutes were taken to finish the whole procedure.

### Image preprocessing

Data preprocessing was carried out by MATLAB2013b (<http://www.mathworks.com/products/matlab/>) and Data Processing & Analysis for Brian Imaging (DPABI) based on SPM8 (<http://www.fifil.ion.ucl.ac.uk/spm/>). The first ten volumes of the scanning session were discarded to allow for T1 equilibration effects. Corrections were performed for the intra-volume acquisition time differences among slices and inter-volume motion effects during the scan. Functional and structural images were co-registered. Structural images were then normalized and segmented into gray matter (Note: gray matter was used as a covariate in statistical comparison between groups), white matter and CSF partitions using the DARTEL technique. A Friston 24-parameter model was used to regress out head motion effects from the realigned data. CSF, white matter, and the global signals as well as the linear trend were also regressed as nuisance covariates. After realigning, slice-timing correction, and co-registration, framewise displacement (FD) was calculated for all resting-state volumes . All volumes with a FD greater than 0.2 mm were regressed out as nuisance covariates. The fMRI data were spatially normalized to a standard EPI template and were resampled to 3×3×3 mm^3^ voxels. Finally, functional images were spatially smoothed with a Gaussian kernel of 6×6×6 mm (full width at half maximum, FWHM).

# References

Cedres, N., Machado, A., Molina, Y., Diaz-Galvan, P., Hernandez-Cabrera, J. A., Barroso, J., et al. (2019). Subjective Cognitive Decline Below and Above the Age of 60: A Multivariate Study on Neuroimaging, Cognitive, Clinical, and Demographic Measures. *J Alzheimers Dis, 68*(1), 295-309. doi:10.3233/JAD-180720

Chen, J., Chen, G., Shu, H., Chen, G., Ward, B. D., Wang, Z., et al. (2019a). Predicting progression from mild cognitive impairment to Alzheimer's disease on an individual subject basis by applying the CARE index across different independent cohorts. *Aging (Albany NY), 11*(8), 2185-2201. doi:10.18632/aging.101883

Chen, J., Duan, X., Shu, H., Wang, Z., Long, Z., Liu, D., et al. (2016a). Differential contributions of subregions of medial temporal lobe to memory system in amnestic mild cognitive impairment: insights from fMRI study. *Sci Rep, 6*, 26148. doi:10.1038/srep26148

Chen, J., Shu, H., Wang, Z., Liu, D., Shi, Y., Zhang, X., et al. (2015). The interaction of APOE genotype by age in amnestic mild cognitive impairment: a voxel-based morphometric study. *J Alzheimers Dis, 43*(2), 657-668. doi:10.3233/JAD-141677

Chen, J., Shu, H., Wang, Z., Zhan, Y., Liu, D., Liao, W., et al. (2016b). Convergent and divergent intranetwork and internetwork connectivity patterns in patients with remitted late-life depression and amnestic mild cognitive impairment. *Cortex, 83*, 194-211. doi:10.1016/j.cortex.2016.08.001

Chen, J., Shu, H., Wang, Z., Zhan, Y., Liu, D., Liu, Y., et al. (2019b). Intrinsic connectivity identifies the sensory-motor network as a main cross-network between remitted late-life depression- and amnestic mild cognitive impairment-targeted networks. *Brain Imaging Behav*. doi:10.1007/s11682-019-00098-4

Dunn, C. J., Duffy, S. L., Hickie, I. B., Lagopoulos, J., Lewis, S. J., Naismith, S. L., et al. (2014). Deficits in episodic memory retrieval reveal impaired default mode network connectivity in amnestic mild cognitive impairment. *Neuroimage Clin, 4*, 473-480. doi:10.1016/j.nicl.2014.02.010

Gu, L. H., Chen, J., Gao, L. J., Shu, H., Wang, Z., Liu, D., et al. (2017). The Effect of Apolipoprotein E epsilon4 (APOE epsilon4) on Visuospatial Working Memory in Healthy Elderly and Amnestic Mild Cognitive Impairment Patients: An Event-Related Potentials Study. *Front Aging Neurosci, 9*, 145. doi:10.3389/fnagi.2017.00145

Hao, L., Wang, X., Zhang, L., Xing, Y., Guo, Q., Hu, X., et al. (2017). Prevalence, Risk Factors, and Complaints Screening Tool Exploration of Subjective Cognitive Decline in a Large Cohort of the Chinese Population. *J Alzheimers Dis, 60*(2), 371-388. doi:10.3233/JAD-170347

Jessen, F., Amariglio, R. E., van Boxtel, M., Breteler, M., Ceccaldi, M., Chetelat, G., et al. (2014). A conceptual framework for research on subjective cognitive decline in preclinical Alzheimer's disease. *Alzheimers Dement, 10*(6), 844-852. doi:10.1016/j.jalz.2014.01.001

Petersen, R. C., Smith, G. E., Waring, S. C., Ivnik, R. J., Tangalos, E. G., & Kokmen, E. (1999). Mild cognitive impairment: clinical characterization and outcome. *Arch Neurol, 56*(3), 303-308. doi:10.1001/archneur.56.3.303

Winblad, B., Palmer, K., Kivipelto, M., Jelic, V., Fratiglioni, L., Wahlund, L. O., et al. (2004). Mild cognitive impairment--beyond controversies, towards a consensus: report of the International Working Group on Mild Cognitive Impairment. *J Intern Med, 256*(3), 240-246. doi:10.1111/j.1365-2796.2004.01380.x

Xue, C., Yuan, B., Yue, Y., Xu, J., Wang, S., Wu, M., et al. (2019). Distinct Disruptive Patterns of Default Mode Subnetwork Connectivity Across the Spectrum of Preclinical Alzheimer's Disease. *Front Aging Neurosci, 11*, 307. doi:10.3389/fnagi.2019.00307

Yan, C. G., Wang, X. D., Zuo, X. N., & Zang, Y. F. (2016). DPABI: Data Processing & Analysis for (Resting-State) Brain Imaging. *Neuroinformatics, 14*(3), 339-351. doi:10.1007/s12021-016-9299-4

Yan, T., Wang, W., Yang, L., Chen, K., Chen, R., & Han, Y. (2018). Rich club disturbances of the human connectome from subjective cognitive decline to Alzheimer's disease. *Theranostics, 8*(12), 3237-3255. doi:10.7150/thno.23772
